# Well Done: Armadillos, Leprosy, and the Meaning of Risk in the Field

**DOI:** 10.4269/ajtmh.26-0583

**Published:** 2026-11-10

**Authors:** Carlos Bastidas-Caldes

**Affiliations:** School of Medicine, Universidad Espíritu Santo, Samborondón, Ecuador

I was sitting beside a fire in a Shuar community in the tropical forests of coastal Ecuador, watching a nine-banded armadillo roast in its own shell. Around me, the scene seemed entirely ordinary. Hunters talked while smoke drifted through the air, and our hosts waited for the meat to cook. Armadillo was familiar food here, even a delicacy.

They cooked it longer and handed it to me, still inside its shell. I looked at the meat and hesitated. It did not look cooked enough. I asked them to put it back over the fire. Their expressions changed. They seemed puzzled by my insistence. I smiled and repeated that I preferred it very well done. What I did not tell them was what had been running through my mind since the animal had been placed over the flames: *Mycobacterium leprae*.

Several years earlier, our research team had begun traveling across Ecuador to investigate whether wild armadillos could harbor *M. leprae*. The work took us through tropical landscapes along the Pacific coast and into the Amazon, often far from conventional sampling sites. Finding armadillos, however, depended less on laboratory expertise than on people who knew these forests intimately. Local hunters knew where the animals lived, when they emerged, and how to find them. Many also hunted armadillos for food, making their knowledge essential to our fieldwork.

One of those journeys brought us to a Shuar community in the tropical forests near the border between Guayas and Azuay Provinces. Their presence there fascinated me. The Shuar are historically associated with the Ecuadorian Amazon, yet this community had settled west of the Andes generations earlier. Geography had changed, but some practices had traveled with them. Hunting wild animals, including armadillos, remained part of everyday life, and what had once been primarily a source of food had also become something visitors could encounter as part of the local experience.

At first, the hunters were reluctant to talk to us. Their caution was understandable. Wildlife hunting in Ecuador is subject to legal restrictions, and conversations about hunting and selling wild meat can therefore be sensitive. We had not come to question or judge these practices; we wanted to understand where armadillos could be found and, eventually, whether they might harbor *M. leprae*. Still, we were outsiders asking about something people did not necessarily want to discuss. With time, brief exchanges became longer conversations. Hunters described where they found armadillos, how they hunted them, and how the animals were prepared. Eventually, we were invited to share the food ourselves.

When the armadillo was finally offered to us, declining did not feel like a simple choice. We had spent time building a relationship with people who had initially been reluctant to speak with us, and sharing food was part of that growing trust. Refusing something our hosts considered a delicacy felt, at least to me, as though it might create a distance we had only just begun to overcome. So, I accepted. Yet accepting the invitation did not silence what I knew. I was a guest at their table, but I was also a microbiologist studying a bacterium for which the animal in front of me was a recognized reservoir.

I was not trying to enjoy an exotic meal. I was trying to get through one without allowing what I knew about *M. leprae* to overwhelm the moment. Asking for the meat to be cooked longer was the only precaution that felt available to me. Perhaps it was partly rational and partly psychological, but each additional minute over the fire made accepting the invitation feel easier. My hosts could not have known what was happening in my mind. To them, my insistence probably reflected an unusual preference for overcooked meat. To me, it was an attempt to reconcile two things that had suddenly become difficult to separate: the trust required to share their food and the scientific knowledge that made me wary of it.

Years later, the laboratory results gave that evening a different meaning. Across Ecuador, we detected *M. leprae* in 9 of the 48 armadillos examined, all of them nine-banded armadillos (*Dasypus novemcinctus*). None of the seven animals sampled in Guayas tested positive. I will never know whether the armadillo placed before me that evening carried *M. leprae*, and there is no reason to assume that it did. But our findings confirmed that the possibility I had been contemplating beside that fire was not merely theoretical: *M. leprae* was circulating in wild armadillos in Ecuador. The results did not tell me what had been on my plate. They changed what that memory meant.

Looking back, what stays with me is not the taste of the armadillo, I can barely remember it, but the difference between what each of us saw that evening. Around the fire, my hosts saw food, tradition, and something worth sharing with a guest. I saw those things too, but scientific knowledge had added another layer: reservoir, pathogen, exposure, risk. There was something almost enviable in their ability to enjoy the meal without those words intruding on the experience. Field epidemiology has taught me that risk is not experienced as a prevalence, a polymerase chain reaction test result, or a transmission pathway; it is filtered through culture, familiarity, trust, and knowledge. We were sitting around the same fire, looking at the same animal, yet experiencing something entirely different. They were sharing a meal. I was calculating a risk. And when they asked how I wanted my armadillo, there was really only one answer I could give: well done.

